# Virtual Screening Analysis and *In-vitro *Xanthine Oxidase Inhibitory Activity of Some Commercially Available Flavonoids

**Published:** 2013

**Authors:** Muthuswamy Umamaheswari, Arumugam Madeswaran, Kuppusamy Asokkumar

**Affiliations:** *College of Pharmacy, Sri Ramakrishna Institute of Paramedical Sciences, Coimbatore-641044, Tamil Nadu, India. *

**Keywords:** Xanthine oxidase, Flavonoids, Binding energy, Enzyme kinetics, Gout

## Abstract

Allopurinol, the xanthine oxidase inhibitor, is the only drug available for the treatment of gout. We examined the xanthine oxidase inhibitory activity of some commercially available flavonoids such asepigallocatechin, acacatechin, myricetin, naringenin, daidzein and glycitein by virtual screening and *in-vitro *studies. The interacting residues within the complex model and their contact types were identified. The virtual screening analysis were carried out using AutoDock 4.2 and *in-vitro *xanthine oxidase inhibitory activity was carried out using xanthine as the substrate. In addition, enzyme kinetics was performed using LineweaverBurkplot analysis. Allopurinol, a known xanthine oxidase inhibitor was used as the standard. The docking energy ofglycitein was found to be -8.49 kcal/mol which was less than that of the standard (-4.47 kcal/ mol). All the selected flavonoids were found to exhibit lower binding energy (-8.08 to -6.03 kcal/ mol) than allopurinol. The docking results confirm that flavonoids showed greater inhibition of xanthine oxidase due to their active binding sites and lesser binding energies compared to allopurinol. This may be attributed to the presence of benzopyran ring in the flavonoids. In the xanthine oxidase assay, IC_50_ value of glycitein was found to be 12±0.86 μg/mL, whereas that of allopurinol was 24±0.28 μg/mL. All the remaining compounds exhibited IC_50_ values ranging between 22±0.64 to 62±1.18 μg/mL. In the enzyme kinetic studies, flavonoids showed competitive type of enzyme inhibition. It can be concluded that flavonoids could be a promising remedy for the treatment of gout and related inflammatory disorders. Further *in-vivo *studies are required to develop potential compounds with lesser side effects.

## Introduction

Drug discovery and development is a complex, long term and interdisciplinary process. It is a multidimensional and sequential process that begins from target identification, lead discovery process, followed by lead optimization and pre-clinical *in-vitro *and *in-vivo *studies (1). Virtual screening of compound libraries has become a standard technology in modern drug discovery pipelines (2). Traditionally, drugs were synthesized from a variety of compounds and screened for its toxicity and biological activities and additionally examined for their pharmacokinetic profile. However, this process is generally time consuming (3). Structure based drug design is becoming a valuable and integral part of drug discovery process, which has been proven to be more effective than the ligand based drug design (4). Studies of interactions between protein domains and ligands are important in virtual screening analysis (5). 

Virtual screening analysis can help in identifying drug targets via bioinformatics tools. They are used to analyze the target structures for possible binding sites, generation of candidate molecules, checking for their drug likeness, docking the molecules with the target, ranking them according to their binding affinities, and further optimization of the molecules to improve binding characteristics (6). Autodock 4.2 is a suite of automated docking tools. It usually starts with the definition of a binding site, in general at a restricted region of the protein. Autodock uses Monte Carlo and Simulated Annealing in combination with Genetic Algorithm which is used for global optimization (7).

Xanthine oxidase (XO) is a highly versatile enzyme that is widely distributed among different species from bacteria to man and within the various tissues of mammals. It is a member of group of enzymes known as molybdenum iron – sulphur flavin hydroxylases (8). It catalyses the oxidation of hypoxanthine to xanthine and then to uric acid, the final reactions in the metabolism of purine bases (9). The accumulation of uric acid in the body is responsible for several diseases and thus it plays a vital role in hyperuraecimia and gout (10). Inherited xanthine oxidase reductase (XOR) deficiency leads to xanthineuria and multiple organ failure syndrome caused by the accumulation of xanthine in different tissues (11).

Xanthine oxidase inhibitors (XOI) are much useful, since they possess lesser side effects compared to uricosuric and anti inflammatory agents (12). Allopurinol is the only clinically available XOI, which also suffers from many side effects such as hypersensitivity syndrome, Steven’s Johnson syndrome and renal toxicity. Thus, itis necessary to develop compounds with XOI activity with lesser side effects compared to allopurinol. Flavonoids and polyphenols have been reported to possess xanthine oxidase inhibitory activity (13).In addition, flavonoids also have anti inflammatory and antitumor properties (14). We thus began our work to look for virtual screening analysis and *in-vitro *xanthine oxidase inhibitory activity of some commercially available flavonoids. 

## Experimental

*Softwares required*

Python 2.7 - language was downloaded from www.python.com, Cygwin (a data storage) c:\program and Python 2.5 were simultaneously downloaded from www.cygwin.com, Molecular graphics laboratory (MGL) tools and AutoDock 4.2 was downloaded from studio visualizer 2.5.5 was downloaded from www.accelerys.com, Molecular orbital package (MOPAC), Chemsketch was downloaded from www.acdlabs.com. Online smiles translatory notation was carried out using cactus.nci.nih.gov/translate/.

*Chemicals required*

Allopurinol, xanthine, xanthine oxidase from bovine milk source and flavonoids such asepigallocatechin, acacatechin, myricetin, naringenin, daidzein, glyciteinwere purchased from Sigma Aldrich, USA. All other drugs and chemicals used in the study were obtained commercially and were of analytical grade.

*Virtual screening analysis*

**Figure 1:**
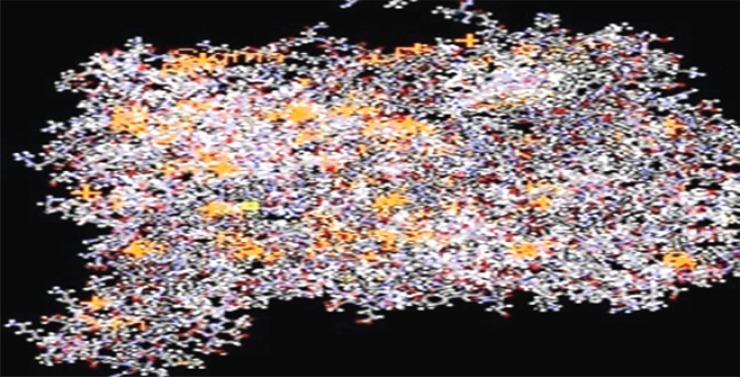
Xanthine oxidase from bovine milk source (3BDJ).

The ligands such asepigallocatechin, acacatechin, myricetin, naringenin, daidzein, glycitein were built using Chemsketch and optimized using “Prepare Ligands” in the Autodock 4.2 for docking studies. Xanthine oxidase model from bovine milk source was downloaded from the RCSB protein data bank (Figure 1). 

The optimized ligand molecules were docked into refined xanthine oxidase model using “Ligand Fit” in the Autodock 4.2 (15). These file preparations were carried out by plugin using scripts from the Autodock Tools package. The ligand score is an automated tool for protein-ligand docking that can define binding site, generate ligand conformations, dock each conformation, save the top docked structures (diverse poses) and apply scoring functions to each docked structure for the best binding mode. The binding sites for these molecules were selected based on the ligand-binding pocket of the templates. For each ligand, 10 poses were generated and scored using Autodock 4.2 scoring functions. Among these poses, the most suitable docking mode for flavone with a high score from consensus scoring functions was finally selected (16 - 18).

**Figure 2:**
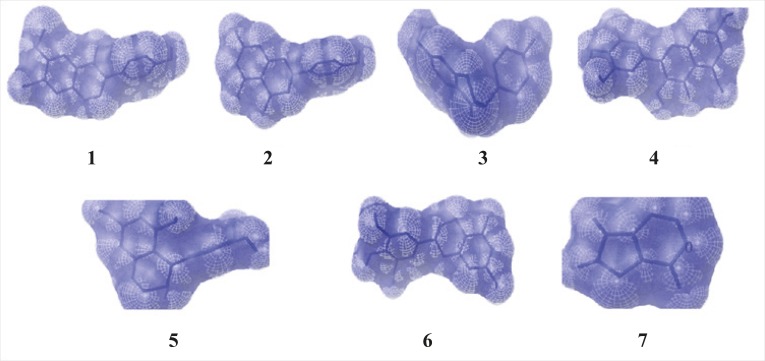
The binding position of ligands to the enzymexanthine oxidase (1 - Glycitein,2 - Naringenin, 3 - Daidzein,4 - Myricitein, 5 -Acacatechin,6 - Epigallocatechin and 7 - Allopurinol)**.**

*In-vitro xanthine oxidase inhibitory activity*

The assay mixture consisted of 1mL of the test compound (5 – 100 μg/mL), 2.9 mL of phosphate buffer (pH 7.5) and 0.1 mL of xanthine oxidase enzyme solution (0.1 units/mL in phosphate buffer, pH 7.5), which was prepared immediately before use. After preincubation at 25 ºC for 15 min, the reaction was initiated by the addition of different concentration (5 – 100 μg/mL) of the substrate solution. The assay mixture was incubated at 25 ºC for 30 min. The reaction was stopped by adding 1 mL of 1 N HCl and the absorbance was measured at 290 nm using an UV spectrophotometer (19, 20). Allopurinol (5 – 100 μg/mL) was used as the standard. The percentage inhibition was calculated by,

Percentage inhibition ={ (A-B) – (C-D)}/(A-B)} X 100 

where A is the activity of the enzyme without the compound, B is the control of A without the compound and enzyme, C and D are the activities of the compound with or without XO respectively. The assay was done in triplicate and IC_50 _values were calculated from the percentage inhibition (21).

*Enzyme kinetics studies*

Lineweaver – Burk plot analysis was performed to determine the mode of inhibition of flavonoids and compared with allopurinol.The assay was carried out in the presence or absence of flavonoids with varying concentrations of xanthine as the substrate, employing the xanthine oxidase assay as mentioned earlier. Lineweaver – Burk transformed values were plotted to determine the mode of enzyme inhibition (22, 23).

## Results and Discussion

*Virtual screening analysis*

**Figure 3:**
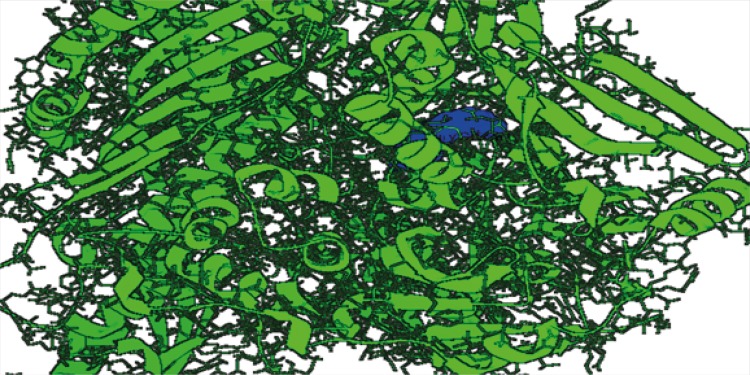
Docked structure of 3BDJ enzyme model (green) withglycitein ligand (blue)**.**

The virtual screening analysis was performed by the use of Autodock 4.2. The docking poses are ranked according to their docking scores and both the ranked list of docked ligands and their corresponding binding poses (24). In Figure 3, docked pose of xanthine oxidase enzyme (green) with glycitein ligand (blue) clearly demonstrates the binding positions of the ligand with the enzyme.

As shown in Table 1, flavonoids showed binding energy ranging from -8.49 kcal/mol to -6.03 kcal/mol. All the selected compounds had lesser binding energy when compared to the standard allopurinol(-4.47 kcal/mol). This proves that flavonoids consist of potential xanthine oxidase inhibitory binding sites when compared to the standard.

**Table 1:** Binding energies of the compounds based on their rank

**COMPOUNDS**	**Binding energies of the compounds based on their rank (kcal/mol)**									
	**1**	**2**	**3**	**4**	**5**	**6**	**7**	**8**	**9**	**10**
Epigallocatechin	-6.03	-5.75	-5.57	-5.40	-5.38	-5.23	-5.19	-4.81	-4.97	-4.93
Acacatechin	-6.22	-6.13	-5.87	-5.79	-5.35	-4.95	-5.96	-5.91	-5.22	-3.99
Myricetin	-7.27	-6.96	-5.09	-7.03	-6.35	-5.86	-5.65	-5.37	-4.78	-4.28
Naringenin	-7.60	-7.40	-7.25	-6.70	-7.18	-7.13	-7.16	-5.79	-5.58	-5.36
Daidzein	-7.54	-7.53	-7.44	-6.83	-5.83	-5.74	-5.63	-5.74	-5.57	-5.49
Glycitein	-8.49	-7.28	-7.20	-6.71	-6.68	-6.66	-6.55	-6.53	-6.46	-5.70
Allopurinol	-4.47	-4.47	-4.46	-4.46	-4.45	-4.20	-4.09	-4.09	-3.99	-3.87

In addition, two other parameters such asinhibition constant (K_i_) and intermolecular energy were also determined. As shown in Table 2, flavonoids showed inhibition constantranging from 596.29 nM to 37.79 μM. All the selected compounds had lesser inhibition constantwhen compared to the standard (529.73 μM). Inhibition constant is directly proportional to the binding energy. We found a decrease in inhibition constant of all the selected flavonoids with a simultaneous decrease in the binding energy. When the binding energy of the compound decreases, there is an increase in activity. Thus, the xanthine oxidase inhibitory activity of the flavonoids were found to be higher compared to allopurinol.

**Table 2:** Inhibition Constant of the compounds based on their rank

**COMPOUNDS**	Inhibition Constant of the compounds based on their rank (μM, mM*, nM**)									
	**1**	**2**	**3**	**4**	**5**	**6**	**7**	**8**	**9**	**10**
Epigallocatechin	37.79	60.80	81.95	110.66	113.88	146.80	157.99	300.11	226.29	242.72
Acacatechin	27.70	31.98	49.96	57.13	119.08	233.44	42.50	46.46	149.99	1.19*
Myricetin	4.67	7.94	184.82	7.02	22.11	52.03	72.20	115.36	315.69	729.56
Naringenin	2.66	3.77	4.81	12.19	5.46	5.94	5.66	57.27	81.19	117.43
Daidzein	2.99	3.02	3.53	9.89	53.26	61.95	74.33	62.28	82.39	94.29
Glycitein	596.29**	4.63	5.31	12.07	12.70	13.02	15.93	16.35	18.52	66.36
Allopurinol	529.73	534.14	541.00	541.30	545.56	830.85	1.01*	1.01*	1.18*	1.45*

As shown in Table 3, flavonoids showed intermolecular energy ranging between -9.68 to -8.01 which was lesser when compared to the standard (-4.47). Intermolecular energy is also directly proportional to the binding energy. We found a decrease in intermolecular energy of all the selected compounds with a simultaneous decrease in the binding energy.This result further proved the xanthine oxidase inhibitory activity of all the selected flavonoids.

**Table 3:** Intermolecular energies of the compounds based on their rank

**COMPOUNDS**	**Inter molecular energies of the compounds based on their rank**									
	**1**	**2**	**3**	**4**	**5**	**6**	**7**	**8**	**9**	**10**
Epigallocatechin	-8.12	-7.84	-7.66	-7.49	-7.47	-7.32	-7.27	-6.89	-7.06	-7.02
Acacatechin	-8.01	-7.92	-7.66	-7.58	-7.14	-6.74	-7.75	-7.70	-7.01	-5.78
Myricetin	-9.36	-9.05	-7.18	-9.12	-8.44	-7.94	-7.74	-7.46	-6.86	-6.37
Naringenin	-8.80	-8.59	-8.45	-7.90	-8.37	-8.32	-8.35	-6.98	-6.77	-6.55
Daidzein	-8.43	-8.43	-8.33	-7.72	-6.73	-6.64	-6.53	-6.63	-6.47	-6.39
Glycitein	-9.68	-8.47	-8.39	-7.90	-7.87	-7.86	-7.74	-7.72	-7.65	-6.89
Allopurinol	-4.47	-4.47	-4.46	-4.46	-4.45	-4.20	-4.09	-4.09	-3.99	-3.87

Docking ofallopurinol in themolybdopterin domain of xanthine oxidase showed the same position and interaction as that of salicylate binding except that Phe 1009 was far away from the bicyclic ring and a hydrogen bonding to Glu 802. We superimposed glycitein with salicylate revealing that bicyclic benzopyranone ring overlapped with salicylate ring, and the phenolic group of the selected flavonoids stretched to the space surrounding with several hydrophobic residues including Phe 1076, Phe 649, Leu 648, Leu 873, and Leu 1014. Several hydrogen bonds and electrostatic interactions were exposed including C7 hydroxyl binding to Glu 1261 via water, C5 hydroxyl close to the guanidium group of Arg 880 and C4 carbonyl interaction with hydroxyl group of Thr 1010. The probabilistic interactions between residues in the microenvironment of binding site to the benzopyran ring of flavonoids decisively prove the xanthine oxidase inhibitory activity by emphasizing its structural properties.

Based on the docking studies, the activity of the selected compounds was in order of

**Figure 4:**
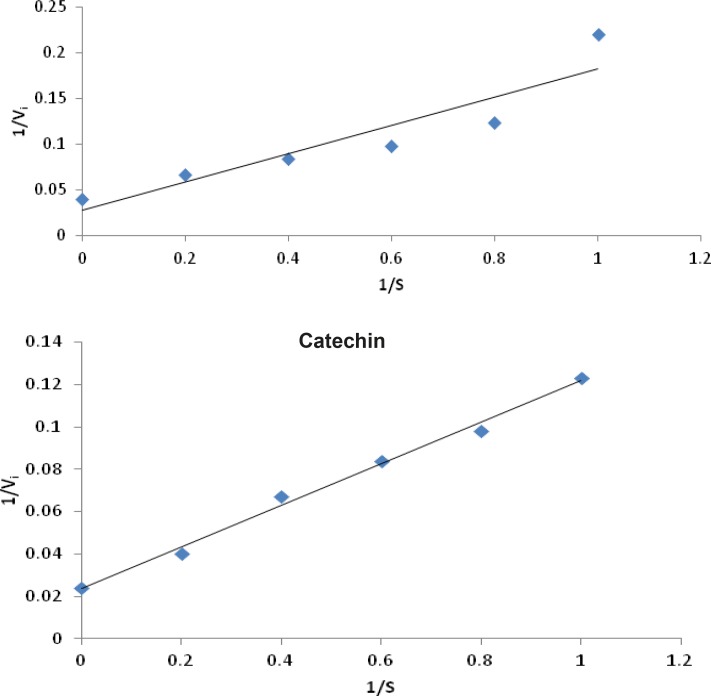
Lineweaver-Burk plot of inhibition of xanthine oxidase by allopurinol and catechin both showing competitive type of enzyme inhibition

**Table 4:** *In-vitro *xanthine oxidase inhibitory activity of the selected compounds

**COMPOUNDS**	**Percentage Inhibition (Mean±SEM)**					**IC**_50_ **μg/mL (Mean±SEM)**
	**Concentration (μg/mL)**					
	5	20	40	60	80	
Epigallocatechin	31.23±0.73	44.53±0.93	54.32±0.64	74.53±1.21	87.54±0.87	36±0.64
Acacatechin	32.00±0.75	39.33±0.92	52.33±0.64	76.00±0.78	85.33±1.26	27±1.16
Myricetin	28.43±0.42	46.76±0.65	58.45±0.54	72.82±0.76	86.72±0.84	26±0.72
Naringenin	33.26±0.65	48.46±0.82	64.73±0.48	82.65±0.65	92.76±0.45	22±0.64
Daidzein	32.87±0.54	46.57±0.62	62.37±0.65	78.65±0.83	87.54±0.76	23±0.75
Glycitein	29.64±0.76	68.76±0.93	75.46±0.98	87.68±0.54	95.63±0.72	12±0.86
Allopurinol	33.33±0.70	38.00±0.86	66.00±1.20	76.67±1.42	97.33±0.92	29±0.28

## Conclusion

The results of the present study clearly demonstrated the xanthine oxidase inhibitory activity of the selected flavonoids by virtual screening analysis and *in-vitro *assay. Virtual screening analysis is actually an added advantage to screen the xanthine oxidase inhibition. The nature of this inhibition, particularly the stronger effect of glycitein,naringenin, daidzeinandmyricetinthan the standard allopurinol, is interesting and merits its further characterization. Further investigations on the above compounds and *in-vivo *studies are necessary to develop potential chemical entities for the prevention and treatment of gout and related inflammatory disorders.
